# Report on adverse events of COVID-19 vaccines in Iran: a comprehensive national prospective longitudinal analysis

**DOI:** 10.3389/fimmu.2025.1504973

**Published:** 2025-02-14

**Authors:** Hamidreza Jamaati, Saeed Karimi, Shahnam Arshi, Seyed Mohsen Zahraei, Yunes Panahi, Fatemeh Nouri, Maryam Hajimoradi, Arman Hasanzade, Majid Mokhtari, Katayoun Tayeri, Atefeh Abedini, Abdolreza Mohamadnia, Payam Tabarsi, Babak Sharif-Kashani, Majid Marjani, Farin Rashid Farokhi, Seyed Mohammad Reza Hashemian, Fatemeh Sadat Hosseini-Baharanchi, Mostafa Norizadeh, Bahamin Astani, Sima Noorali, Farnaz Ahmadi, Shadi Shafaghi, Fariba Ghorbani

**Affiliations:** ^1^ Chronic Respiratory Disease Research Center, National Research Institute of Tuberculosis and Lung Disease, Shahid Beheshti University of Medical Science, Tehran, Iran; ^2^ Department of Ophthalmology, Torfeh Medical Center, Shahid Beheshti University of Medical Sciences, Tehran, Iran; ^3^ Center for Communicable Disease Control, Ministry of Health and Medical Education, Tehran, Iran; ^4^ Chemical Injuries Research Center, Baqiyatallah University of Medical Sciences, Tehran, Iran; ^5^ Lung Transplantation Research Center, National Research Institute of Tuberculosis and Lung Diseases (NRITLD), Shahid Beheshti University of Medical Sciences, Tehran, Iran; ^6^ Tracheal Diseases Research Center, National Research Institute of Tuberculosis and Lung Diseases, Shahid Beheshti University of Medical Sciences, Tehran, Iran; ^7^ Department of Pulmonary and Critical Care Medicine, Loghman Hakim Hospital, Shahid Beheshti University of Medical Sciences, Tehran, Iran; ^8^ Clinical Tuberculosis and Epidemiology Research Center, National Research Institute for Tuberculosis and Lung Disease (NRITLD), Shahid Beheshti University of Medical Sciences, Tehran, Iran; ^9^ Department of Cardiology, Lung Transplantation Research Center, National Research Institute of Tuberculosis and Lung Diseases, Shahid Beheshti University of Medical Sciences, Tehran, Iran; ^10^ Chronic Kidney Disease Research Center, Shahid Beheshti University of Medical Sciences, Tehran, Iran; ^11^ Department of Biostatistics, School of Public Health, Iran University of Medical Sciences, Tehran, Iran; ^12^ Department of Biotechnology, Faulty of Advanced Sciences and Technology, Tehran Medical Sciences, Islamic Azad University, Tehran, Iran

**Keywords:** COVID-19 vaccination, Iran, adverse events, complication, safety, vaccination

## Abstract

**Introduction:**

The global coronavirus disease 2019 (COVID-19) pandemic necessitated urgent vaccine development, raising concerns about potential vaccine adverse events (AEs).

**Methods:**

In this prospective cohort study, conducted from February 2021 to December 2022, all individuals across Iran who received any COVID-19 vaccine dose and reported adverse events were investigated. Our aim was to evaluate these AEs based on the vaccine types, patients’ age and types of AES, and provide a comprehensive analysis.

**Results:**

In Iran, 155 million COVID-19 vaccine doses were administered, with Covilo (Sinopharm) being the most commonly vaccine administered (80.35%). Adverse events predominantly affected individuals aged 40-70 (45%). A total of 86,275 adverse events were recorded, with 92.7% classified as non-serious and 6 299 (7.3%) serious and among the serious cases, 279 were confirmed to be vaccine-related, with 46 resulting in fatalities and 233 requiring hospitalizations. The incidence of serious AEs was 0.41 per 10,000 doses. Serious AEs were more frequently associated with Sputnik V and Vaxzevria (AstraZeneca), with 0.73 and 0.64 cases per 10,000 injections, respectively. Coagulation and thrombosis disorders were the most common serious AEs (29%), followed by neurological (24.7%) and cardiovascular AEs (15.8%).

**Discussion:**

In conclusion, the AEs of COVID-19 vaccination were primarily mild and transient, while serious AEs remained exceptionally rare.

## 1 Introduction

The global impact of infection with the Severe Acute Respiratory Syndrome Coronavirus 2 (SARS-CoV-2), known as coronavirus disease 2019 (COVID-19), has caused over 7.06 million deaths worldwide and 146,000 mortalities in Iran as of May 10, 2023 (1). There was an urgent need to develop a vaccine to overcome this crisis and subsequent medical, economic, and social consequences. Therefore, various vaccines have been developed globally in 4 categories based on their production technology: inactivated virus, viral vector, protein-based, and nucleic acid-based (2). Approximately 13.3 billion vaccine doses have been administered, and their effectiveness in mitigating COVID-19 complications has been evaluated (1, 3–5).

Enhancing vaccination policy and maintaining public trust required transparently identifying and announcing the adverse events (AEs) of vaccines by active and passive COVID-19 vaccine safety surveillance as two complementary approaches for monitoring the safety and efficacy of vaccines after they are administered to the population (6). Passive surveillance relies especially on vaccine recipients to report adverse events following immunization (AEFI) voluntarily which this system is simple and cost-effective and covers a wide population and can detect rare adverse events. In active surveillance approach, vaccinated individuals are followed over time to monitor for adverse event. It involves proactively seeking out information about adverse events by directly contacting individuals or analyzing structured datasets. Investigations have revealed that fatigue, headache, myalgia, fever, and injection site pain are the most observed AEs following vaccination. In addition, rare AEs have been reported, including thrombosis, myocarditis, and Guillen-Barre syndrome (2, 7, 8). It is noteworthy that these AEs usually exhibited mild-to-moderate severity, indicating the overall safety of COVID-19 vaccines (3, 9, 10).

The COVID-19 vaccination in Iran started in February 2021, and by the end of 2022, 155 million vaccine doses had been administered (1), consisting of 3 distinct platforms and 10 brands. These included inactivated virus platform (Covilo, Covaxin, Barekat, and FAKHRAVAC), adenoviral platform (Sputnik V and Vaxzevria), and recombinant protein platform (PastoCovac, SpikoGen, Razi-Cov-Pars, and Nora). Covilo and PastoCovac, considered safe for individuals aged 5 to 18 (11), were authorized in Iran in March 2021 and October 2021, respectively, and both were administered in two doses (12–14). Moreover, Covilo and Vaxzevria were considered safe in pregnant women (15–17).

In alignment with the World Health Organization (WHO) recommendations to perform Active Vaccine Safety Surveillance (AVSS), Iran obtained performance approval in 2010. The AVSS was established under the supervision of the Ministry of Health and Medical Education (MoHME) and received WHO support across 7 medical universities led by Shahroud University of Medical Sciences. Any post-vaccination medical event is classified as an adverse event (AE), and tracked for 30 days, or up to 6 weeks for conditions like Guillain-Barre syndrome. Additionally, those adverse events resulting in hospitalization or death are classified as serious AEs. Furthermore, it was examined whether the AEs are related to vaccination or not. Meanwhile, several studies have been conducted in Iran, but to this date, none has studied all the COVID-19 vaccines administered nationally (6, 18). Therefore, the purpose of this study was to investigate all reported AEs following COVID-19 vaccine administration, evaluate these AEs based on their respective platforms, and conduct a comparative analysis among them.

## 2 Materials and methods

### 2.1 Design and setting

In this cohort study, individuals who had received at least one dose of the COVID-19 vaccine and reported any adverse events, regardless of their causal relation, were enrolled. Data were collected nationwide from February 2021 to December 2022.

### 2.2 Data sources

All post-vaccination medical occurrences, irrespective of their relation to the vaccine, whether clinical or paraclinical, were considered adverse events in this study. Adverse events were documented for up to 30 days post-vaccination, extended to 6 weeks for cases like Guillain-Barré syndrome to capture delayed onset events. Adhering to standard procedures, Iran’s medical universities’ deputy health department systematically documented and reported all the observed adverse events associated with COVID-19 vaccine administration. These records were investigated by the respective universities and National Committees, overseen by the MoHME. Specifically, the committees included representatives from 7 medical universities led by Shahroud University of Medical Sciences.

To investigate the reported adverse events, all cases were initially evaluated by a physician specializing in the field related to the adverse events. Then, all documents, records, and the patient’s clinical file were sent to the evaluation committee at the Ministry of Health. Further evaluations were conducted by the committee comprising vaccine experts and specialists from various clinical and paraclinical fields.

Subsequently, serious AEs were classified into six groups: 1) vaccine-related reaction (related), subcategorized as certainly related, probably related, and possibly related, 2) unknown, 3) reaction without simultaneous connection, 4) unclassifiable, 5) vaccination error (program error), and 6) anxiety-related reactions.

Following the investigation by the university vaccine adverse events committees, substantive AEs were submitted to the Department of Vaccine-Preventable Maladies to be considered and presented to the National Committee. Accordingly, the National Committee’s weekly meetings on the Coronavirus vaccine adverse events, led by the Deputy of Treatment of Ministry of Health, facilitated the final evaluation of serious AEs. The outcomes were subsequently communicated to the universities.

The present study involved an evaluation of cases evaluated and classified by the National Committee. The final diagnosis of AEs was used to categorize the cases into 5 groups: coagulation disorders and thrombosis (e.g., deep vein thrombosis, pulmonary embolism, and thrombotic thrombocytopenic purpura), neurological disorders (such as Guillain-Barre syndrome, acute disseminated encephalomyelitis, seizures, optic neuritis, Bell’s palsy, etc.), cardiovascular disorders (encompassing myocardial infarction, coronary artery disease, congestive heart failure, myocarditis, pericarditis, etc.), cerebrovascular accidents (such as stroke, intracranial hemorrhage, sinus thrombosis, vasculitis, etc.), and other AEs (including hepatitis, polyserositis, hypertension, arthritis, allergy, anaphylaxis, angioedema, glomerulonephritis, etc.).

### 2.3 Ethical considerations

This study obtained approval from the Iran National Committee for Ethics in Biomedical Research (ethics code: IR.NRITLD.REC.1402.089) and adhered to ethical guidelines of the Declaration of Helsinki.

### 2.4 Data gathering and statistical analysis

Based on AVSS system that involves proactive and systematic monitoring of AEs following vaccination, all post-vaccination medical occurrences were tracked for up to 30 days, or up to 6 weeks for specific conditions such as Guillain-Barré syndrome. The AVSS process includes the structured reporting and classification of AEs including case identification, local evaluation and causality assessment. Then, all relevant data were gathered and recorded in the data were collected in Microsoft Excel (version 2016) and subsequently imported into SPSS (version 27; SPSS Inc., Chicago, IL, USA) for analysis. Categorical variables were reported as percentage (%), and quantitative variables were presented as mean ± standard deviation (SD). In case of non-parametric data median and Interquartile Range (IQR) were also reported.

The statistical analysis of serious AE cases, classified by the National Committee as related AEs, was performed using the one-way analysis of variance (ANOVA) for quantitative variables and the chi-square test for nominal variables. Also, logistic regression was performed to estimate the odds ratio (OR) of improvement regarding age, vaccine platform, and type of AEs. The results were reported as mean difference (MD), OR, 95% Confidence interval (CI), and *P*-value.

Additionally, according to the National Committee, the Incidence Index of Related Serious AEs defines the amount of serious adverse events related to vaccines that occur for every 100,000 doses (Equation 1).


(1) $$\begin{array}{c} Incidence\;Index\\ =\left(Number\;of\;Serious\;Adverse\;Events\right)/\\ \left(Total\;Number\;of\;Vaccine\;Doses\;Administered\right)\times 100,000 \end{array}$$

Also, mortality incidence index of AEs was defined as the amount of death per 100 000 vaccine injections (Equation 2). This statistic offers a standardized assessment of the relative frequency of such occurrences and makes it easier to compare vaccines with different rates of use.


(2) $$\begin{array}{c} Mortality\;Incidence\;Index\\ =\left(Number\;of\;Deaths\;due\;to\;Adverse\;Events\right)/\\ \left(Total\;Number\;of\;Vaccine\;Doses\;Administered\right)\times 100,000 \end{array}$$

## 3 Results

### 3.1 COVID-19 Vaccine frequency distribution in Iran, December 2022

The overall first-dose vaccination rate across all age groups was 77.5%, while the second-dose rate was 69.7%. In the population of children aged 5 to 12 years, approximately 26.6% received the first vaccine dose, with 14.6% undergoing the second-dose vaccination. The COVID-19 vaccination rate was higher among children aged 12 to 18 years old, reaching 95.9% for the first dose and 83.3% for the second dose. For individuals over 18 years old, the vaccine frequency distribution was 91.7% for the first dose, 84.2% for the second dose, and 46.5% for the third dose. The distribution of the administered COVID-19 vaccine types was as follows: Covilo (80.35%), Vaxzevria (9.32%), Barekat (5.96%), PastoCovac (1.88%), SpikoGen (1.17%), Razi-Cov-Pars (0.14%), Sputnik V (0.89%), Covaxin (Bharat Biotech) in (0.17%), FAKHRAVAC (0.05%), and Nora (0.05%).

**Figure 1:**
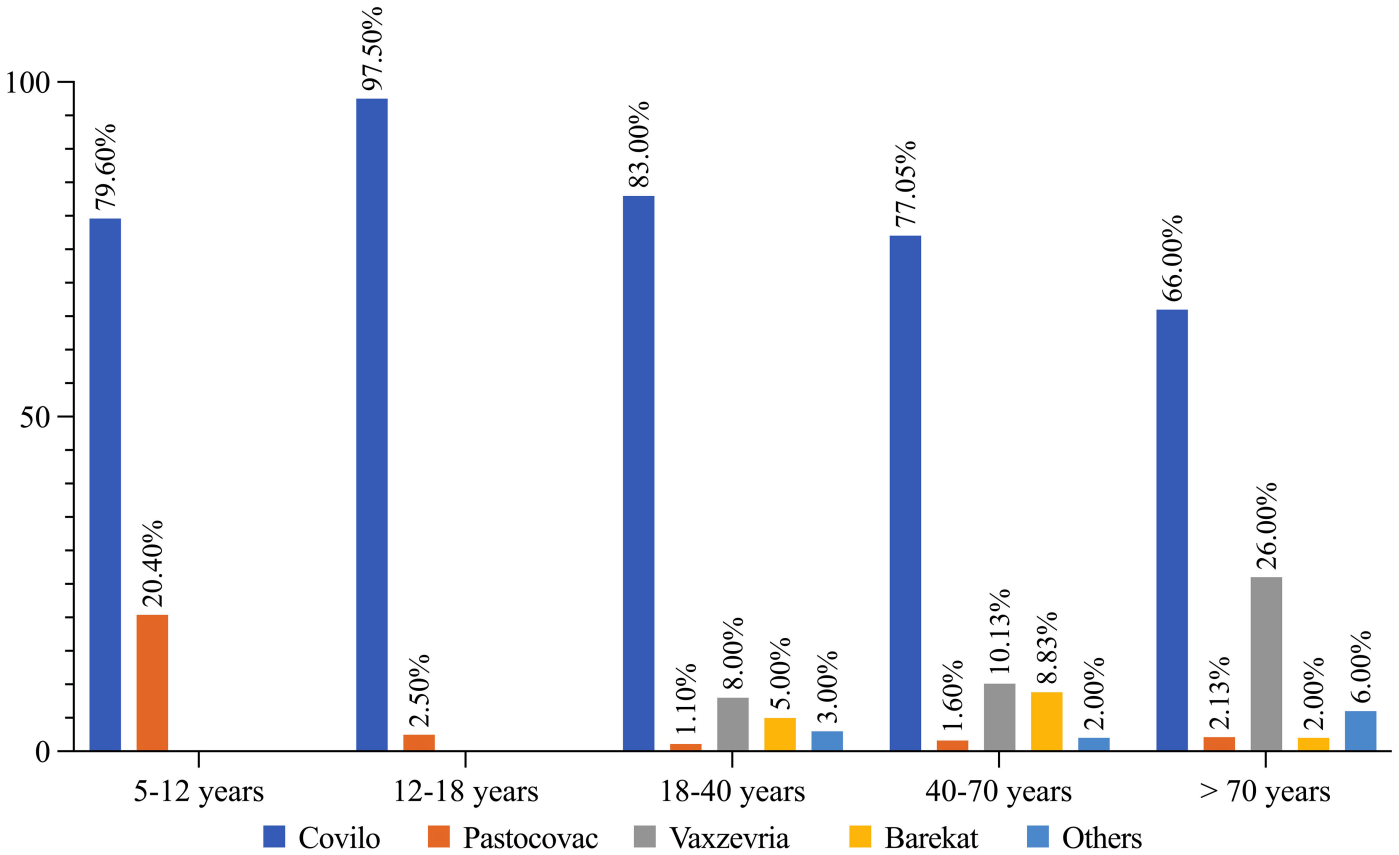
COVID-19 vaccine distribution based on different age categories.

Examining the vaccine distribution across age groups demonstrated the highest injection rate of Covilo in various groups (**Figure 1**).

### 3.2 Total recorded AEs

The overall number of recorded AEs was 86275, comprising 79 976 (92.7%) non-serious and 6 299 (7.3%) serious AEs, of which 592 were classified as relevant in university committees and eventually 279 cases were confirmed as related by National Committees (**Table 1**). The overall incidence of serious vaccine-related AEs, regardless of the causal relationship, was 5.6 cases per 10 000 doses (0.056%). The rates of serious AEs for different types of vaccines were as follows:

**Table 1:** Demographic patterns of administered doses and related AEs by vaccine platform and brand.

Platforms		Adenoviral platform		Inactivated virus platform				Recombinant protein platform			
Vaccines		Sputnik V	Vaxzevria	Covilo	Barekat	FAKHRAVAC	Covaxin	Pastocovac	Razi	SpikoGen	Noora
**Total number of vaccination doses**		1,382,682	14,431,047	124,423,816	9,231,170	97,327	259,772	2,918,006	222,999	1,811,438	74,269
Age^*^	**<12y** **N (%)**	0 (0%)	0 (0%)	2,588,016 (2.08)	0 (0%)	0 (0%)	0 (0%)	657,427 (22.53)	0 (0%)	0 (0%)	0 (0%)
	**12-18y** **N (%)**	0 (0%)	0 (0%)	11,384,779 (9.15)	0 (0%)	0 (0%)	0 (0%)	296,469 (10.16)	0 (0%)	0 (0%)	0 (0%)
	**18-40y** **N (%)**	745,681 (53.93)	5,098,489 (35.33)	52,855,237 (42.48)	3,170,907 (34.35)	53,773 (55.25)	171,164 (65.89)	686,607 (23.53)	112,057 (50.25)	798,923 (44.10)	32,069 (43.18)
	**40-70y** **N (%)**	516,846 (37.38)	6,685,904 (46.33)	50,901,783 (40.91)	5,826,714 (63.12)	38,279 (39.33)	85,517 (32.92)	1,061,862 (36.39)	97,317 (43.64)	846,026 (46.70)	37,625 (50.66)
	**≥70 y** **N (%)**	120,155 (8.69)	2,646,654 (18.34	6,694,001 (5.38)	233,549 (2.53	5,275 (5.42	3,091 (1.19	215,641 (7.39	13,625 (6.11	166,489 9.19	4,575 6.16
Sex^*^	**female**	671,560 (49%)	6,509,212 (45%)	63,984,656 (51%)	4,330,522 (47%)	42,208 (43%)	119,715 (46%)	1,386,395 (48%)	86,846 (39%)	833,290 (46%)	23,748 (32%)
	**male**	711,122 (51%)	7,921,835 (55%)	60,439,160 (49%)	49,00,648 (53%)	55,119 (57%)	140,057 (54%)	1,531,611 (52%)	136,153 (61%)	978,148 (54%)	50,521 (68%)
Relevant AEs^**^	**cardiovascular complications per total N (PMP)**	3 (2.16)	8 (0.55)	29 (0.23)	5 (0.54)	0	0	0	1 (4.48)	0	0
	**Neurological per total N (PMP)**	8 (5.78)	15 (1.03)	42 (0.33)	3 (0.32)	0	0	1 (0.34)	0	0	0
	**Thrombosis per total N (PMP)**	1 (0.72)	34 (2.35)	41 (0.32)	2 (0.21)	0	0	0	0	1 (0.55)	0
	**Cerebrovascular per total N (PMP)**	0	10 (0.69)	19 (0.15)	2 (0.21)	0	0	0	0	0	0
	**Other serious complications per total N (PMP)**	0	27 (1.87)	24 (0.19)	1 (0.1)	0	0	1 (0.34)	0	0	0

*Comparisons across different platforms revealed a *p*-value of <0.001 for age and sex.

**Comparisons across different platforms revealed a *p*-value of 0.33 for AEs.

PMP, Per Million Populations; AEs, Adverse Events.

Sputnik V (363 cases), Covaxin (337 cases), Vaxzevria (8.51 cases), SpikoGen (5.9 cases), Covilo (3.5 cases), Barekat (4.3 cases), Razi-Cov-Pars (2.8 cases), PastoCovac (3.1 cases), Nora (1.8 cases), and FAKHRAVAC (2.4 cases) per 10 000 injections.

**Figure 2:**
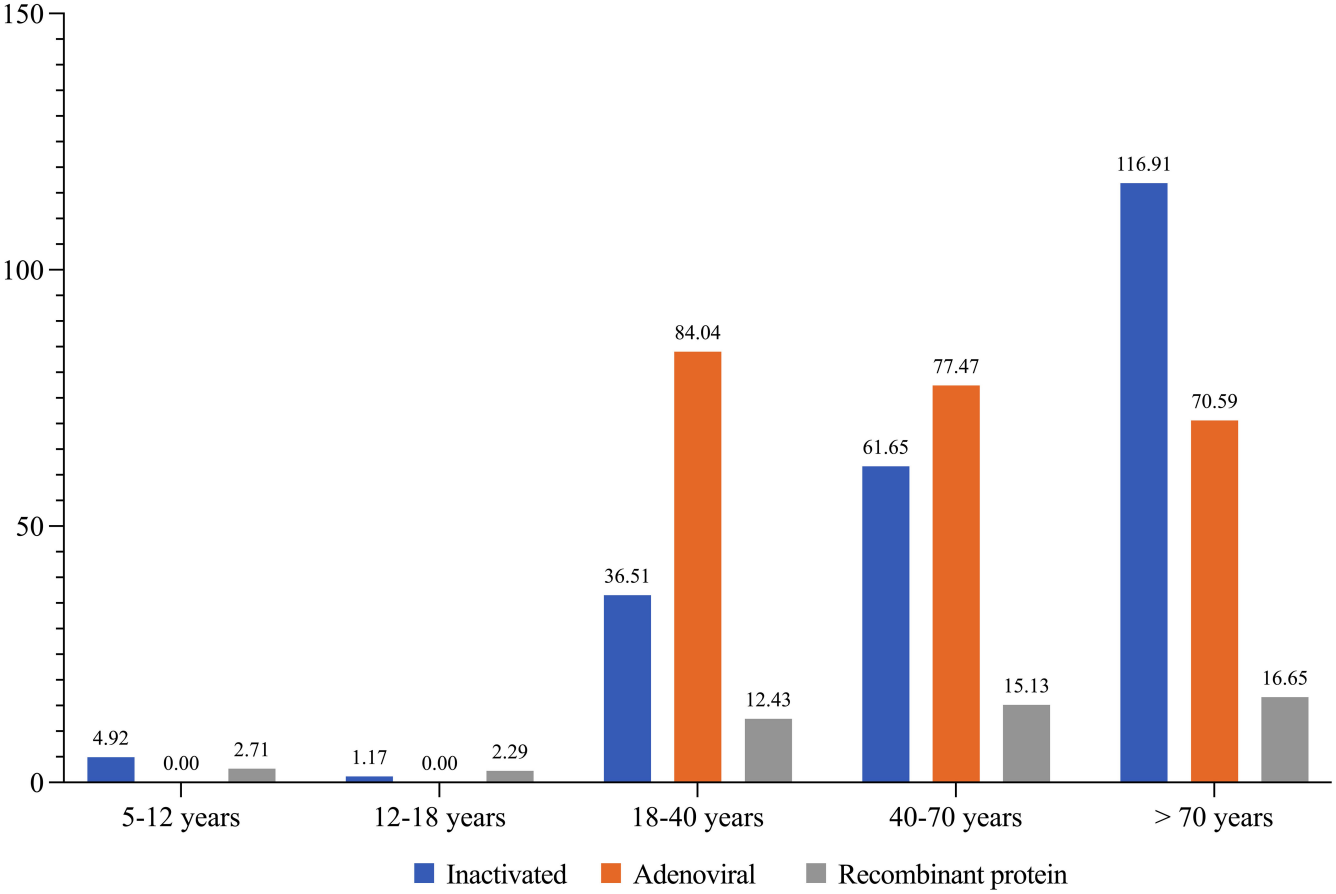
The incidence of total AEs per 10 000 doses of injected vaccine by age groups.

Age-linked vaccine AEs exhibited a consistent pattern, with the most significant incidences observed within the ages of 18-40 (29.4%) and 40-70 years (45%). Meanwhile, age groups 5-12 (1.6%), 12-18 (2.4%), and above 70 years (21.4%) showed varying rates, spanning various vaccines (**Figure 2**).

### 3.3 Non-serious AEs

The overall number of non-serious AEs of the COVID-19 vaccine was 121 712. The most reported non-serious AEs included flu-like symptoms (30%), low-grade fever (25%), localized symptoms (14%), and joint pain (12%). Less reported non-serious AEs were fever above 38.5°C, diarrhea, vomiting, chills, fainting, allergic reactions, dyspnea, dry cough, lymphadenitis, skin rash, tachycardia, eye redness, increased blood pressure, and low blood pressure.

### 3.4 Serious AEs

Regarding the distribution of serious AEs based on the vaccine platform, Covilo, which constitutes 80.35% of the total vaccines administered, accounts for 55.8% of serious AEs. Vaxzevria, representing 9.32% of the total, is associated with 33.8% of serious AEs. Barekat, making up 5.96% of the total doses, accounts for 4.8% of serious AEs. Sputnik V, at 0.89% of the total doses, is linked to 4.3% of serious AEs. PastoCovac, representing 1.88% of the total, accounts for 0.7% of serious AES. Razi-Cov-Pars, comprising 0.14% of the total doses, is associated with 0.4% of serious complications, while SpikoGen, at 1.17% of the total doses, also accounts for 0.4% of serious AEs. Therefore, 60.3% of all serious AEs were associated with the inactivated vaccine platform, which accounted for 86.31% of the total vaccines administered (80.35% from Covilo and 5.96% from Barekat). On the other hand, 38% of serious AEs were associated with the adenoviral platform, which accounted for a combined 10.21% of vaccines administered and the recombinant protein platform, accounted for 3.19% of vaccines administered and 1.5% of serious AEs.

Among serious AEs, 273 were thrombosis AEs, and a statistically significant male predominance in thrombosis occurrence was observed, with 171 cases (62.6%) reported in men and 102 cases (37.4%) in women (*p*-value = 0.004). The incidence of thrombosis exhibited a dose-dependent trend. 151 cases (56.8%) occurred following the first dose, 96 cases (36.1%) after the second dose, 19 cases (7.1%) after the third dose (*p*-value = 0.32).

#### 3.4.1 Serious AEs classified by the National Complication Committee

The reported incidence of serious AEs attributed to vaccination by the National Complication Committee was 0.41 per 10 000 vaccine doses across all types of vaccines. Out of 592 AEs classified within the National Committee, 279 cases considered related (46 cases died and 233 cases were hospitalized), 259 were simultaneous or irrelevant cases, 2 cases were unknown, 44 cases were unclassifiable (due to insufficient documentation), 6 cases were anxiety reactions caused by the injection, and 2 cases were vaccination errors.

In total, 125 out of 592 cases resulted in death, of which 46 cases were considered related (7.77%), and 467 out of 592 cases resulted in hospitalization, of which 233 cases were defined as related to the vaccine (39.36%).

Regarding the serious AEs reported per 10,000 injections related to each vaccine, 0.73 cases were reported for Sputnik V, 0.64 cases for Vaxzevria, 0.53 cases for Barekat, 0.38 cases for Covilo, 0.35 cases for Covaxin, 0.21 cases for FAKHRAVAC, 0.07 cases for SpikoGen, 0.04 cases for PastoCovac, and 0.09 cases Razi-Cov-Pars. No serious AEs related to the Nora vaccine were reported.

In terms of serious AEs types, out of 279 cases, 29% (81 cases) were coagulation disorders and thrombosis, 24.7% (69 cases) were neurological AEs, 15.8% (44 cases) were cardiovascular AEs, 11.1% (31 cases) were cerebrovascular accidents, and 19.4% (54 cases) were classified into other etiologies.

**Figure 3:**
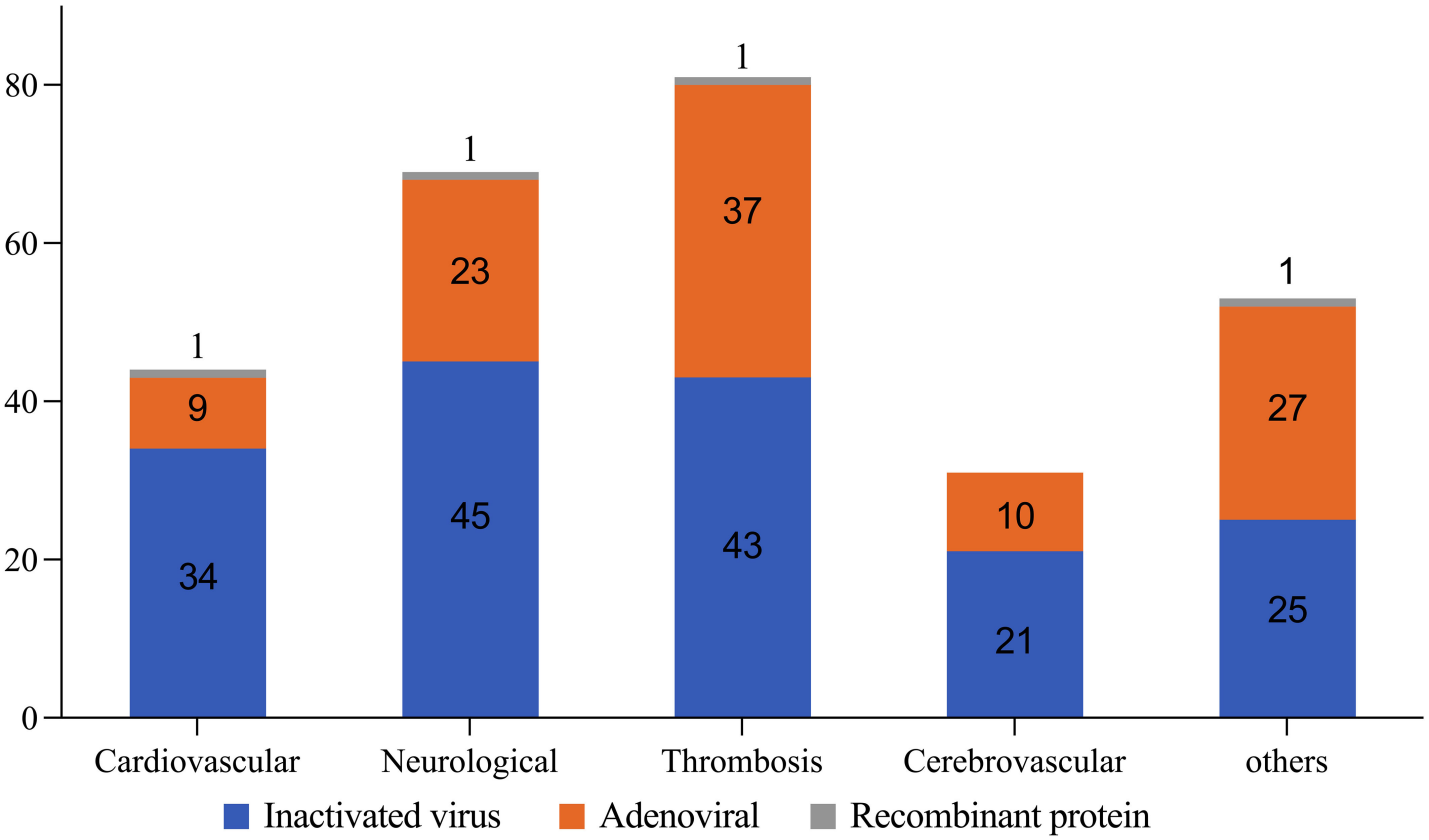
Total serious related AEs types based on different vaccine platforms.

Regarding the hospitalized related cases (233 cases), 28% neurological AEs, 27% coagulation disorders and thrombosis, 21% other etiologies, 15% cardiovascular AEs, and 9% cerebrovascular accidents were observed (**Figure 3**).

A significant age disparity (*P*-value: 0.001) was observed among patients across different serious AE classifications. Patients with cerebrovascular accidents had a mean age of 60.8 ± 17.1 years, while the mean age of those with coagulation and thrombosis was 49.5 ± 21.4, in neurological AEs 42.7 ± 19.2, and in cardiovascular 49.5 ± 16.5 years. Similarly, a significant difference was observed in frequency of the serious AEs regarding sex distribution (*P*-value: 0.003) where 53.1% of coagulation and thrombosis, 56.5% of neurologic, 72.7% of cardiovascular AEs, and 48.4% of cerebrovascular accidents were reported in men.

#### 3.4.2 Incidence index of related serious AEs

**Figure 4:**
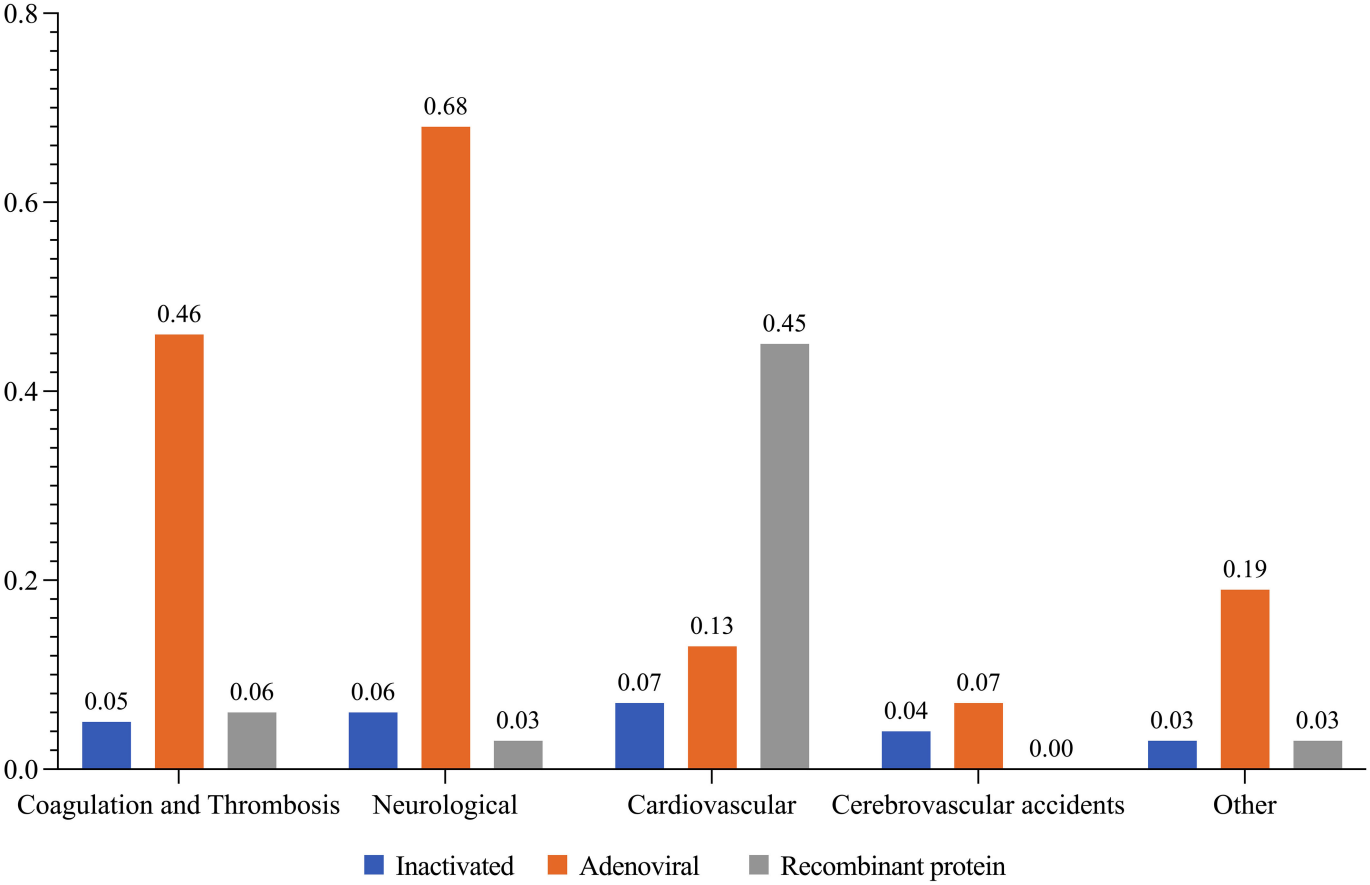
The incidence index of related serious AEs per 100 000 injections by vaccine platforms.

The incidence index of all vaccine related serious AEs per 100,000 administrations were reported as follows: 0.18 in total, Sputnik V 0.87, Vaxzevria 0.65, Razi-Cov-Pars 0.45, Barekat 0.14, Covilo 0.12, PastoCovac 0.07, and SpikoGen 0.06. Accordingly, the incidence index of coagulation disorders and thrombosis were as follows: 0.05 in total, Vaxzevria 0.24, Sputnik V 0.22, SpikoGen 0.06, Covilo 0.03, and Barekat 0.02. Neurological AEs had a total incidence index of 0.04 per 100,000, Sputnik V 0.58, Vaxzevria 0.1, PastoCovac 0.03, Covilo 0.03, and Barekat 0.03. Cardiovascular AEs were 0.03 in total, Razi-Cov-Pars 0.45, Sputnik V 0.07, Vaxzevria 0.06, Barekat 0.05, and Covilo 0.02. The incidence index of cerebrovascular accidents was 0.02 in total, Vaxzevria 0.07, Barekat 0.02, and Covilo 0.02, while AEs with other etiologies had a total incidence of 0.03, Vaxzevria 0.19, Barekat 0.02, and Covilo 0.02 (**Figure 4**).

### 3.5 Thrombosis-related AEs

Out of 155 million administered COVID-19 doses, 279 serious AEs were confirmed as vaccine-related among the reported cases, accounting for only 0.00018% of cases. Among these 279 cases, 136 were thrombotic AEs (48.7% of related serious AEs and 0.000087% of all administrations), and 143 cases were non-thrombotic AEs (51.3% of related serious complications and 0.000092% of all administrations).

In total, thrombotic AEs were related to Covilo in 54% of cases, Vaxzevria in 36%, Barekat in 5%, Sputnik V in 3%, SpikoGen in 1%, and Razi-Cov-Pars in 1%. Women constituted 44% of these cases. Thrombotic AEs occurred in 56% of people after the first dose, 32% after the second dose, and 12% after the third dose. In terms of age distribution, 47% of cases with thrombotic AEs were aged between 40 and 70 years, 26% were aged between 18 and 40 years old, 24% were over 70 years old, and only 3% were aged between 12 and 18 years old.

The incidence index of related thrombotic AEs per 100 000 injections by the vaccine types were as follows: 0.09 in total, Razi-Cov-Pars 0.45, Vaxzevria 0.34, Sputnik V 0.29, Barekat 0.08, SpikoGen 0.06, and Covilo 0.06. Mortality among these 136 thrombotic cases was 25%. The breakdown by sex was 44% women and 55% men.

### 3.6 Non-thrombotic AEs with the associated classification

There were 143 reported cases of non-thrombotic AEs, with 50.5% of them involving women. In terms of age distribution, 39.2% of cases were in the age group of 40 to 70 years, 38.4% in the age group of 18 to 40 years old, 12.6% were over 70 years old, 7.7% in the age group of 12 to 18 years, and 2.1% were in the age group of 5 to 12 years old. These AEs occurred after the first dose of vaccination in 57% of cases, 31% after the second dose, and 12% following the injection of the third dose. Regarding the vaccine type, 58% of these cases were associated with Covilo, 31% with Vaxzevria, 6% with Sputnik V, 4% with Barekat, and 1% with the PastoCovac vaccine. Accordingly, the incidence index of related non-thrombotic AEs per 100 000 injections by vaccine was as follows: 0.09 in total, Sputnik V, 0.58, Vaxzevria 0.31, PastoCovac 0.07, Covilo 0.07, Barekat 0.06. In this group, 92% of cases recovered, while 8% of cases resulted in death.

### 3.7 Mortality in related cases classified by the National Committee

According to the National Committee, there were 46 COVID-19 vaccine related deaths, of which 39.1% were attributed to coagulation disorders and thrombosis, 21.7% to cardiovascular AEs, 19.6% to cerebrovascular accidents, 10.9% to other etiologies (including polyserositis, ischemic pancreatitis, hepatitis, polyserositis, and anaphylaxis), and 8.7% to neurological AEs. An evaluation of mortality across groups revealed a significant difference (*P*-value: 0.006), with mortality rates of 22.2% in coagulation and thrombosis AEs, 22.7% in cardiovascular AEs, 29.0% in cerebrovascular accidents, 5.8% in neurologic disorders, and 9.3% in other AEs.

Consequently, the odds of survival in related serious AEs cases, compared to coagulation and thrombosis AEs, are as follows: for neurologic disorders (OR: 4.64, 95%CI: 1.49-14.48, *P*-value: 0.008), for cardiovascular problems (OR: 0.97, 95%CI: 0.40-2.33, *P*-value: 0.94), for cerebrovascular accidents (OR: 0.69, 95%CI: 0.27-1.78, *P*-value: 0.45), and for other AEs (OR: 2.80, 95%CI: 0.97 - 8.07, *P*-value: 0.05) (**Table 2**).

**Table 2:** Evaluation of mortality details in patients with serious AEs based on AE classes, patient demographics, and vaccine platforms.

	Mortality Rate	Odds of Survival^*^	95% CI	*P*-Value
AEs Classification				
Coagulations and thrombosis (%)	22.2	Reference	–	–
Cardiovascular (%)	22.7	0.97	0.4 – 2.33	0.94
Cerebrovascular (%)	29.0	0.69	0.27 – 1.78	0.45
Neurologic (%)	5.8	4.64	1.49 – 14.48	0.008
Other (%)	9.3	2.80	0.97 – 8.07	0.05
Vaccine Platforms				
Inactivated Virus (%)	13.1	Reference	–	–
Recombinant Protein (%)	50.0	0.15	0.20 – 1.12	0.06
Adenoviral (%)	20.8	0.57	0.30 – 1.10	0.09
Sex				
Male (%)	15.0	Reference	–	–
Female (%)	18.2	0.88	0.65 – 1.21	0.52
	Mean (SD)	Mean Difference	95% CI	*P*-Value
Age				
Survived group (years)	47.0 (20.0)	Reference	–	–
Mortality (years)	52.0 (19.6)	4.96	-1.38 – 11.3	0.12

*The odds of survival were reported compared to the reference group, which is specified.

CI, Confidence Interval.

Furthermore, 45.7% of mortalities were related to Covilo, 43.4% to Vaxzevria, 4.3% to Sputnik V, 2.2% to Barekat, 2.2% to PastoCovac, and 2.2% to Razi. Based on vaccine platforms, 13.1% of patients with related serious AEs in the inactivated platform died. However, the recombinant protein platform had a 50.0% mortality rate in related serious AEs, with odds of survival compared to the inactivated platform (OR: 0.15, 95%CI: 0.20 - 1.12, *P*-value: 0.06), and adenoviral platforms had a 20.8% mortality rate, indicating OR: 0.57, 95%CI: 0.30 -1.10, *P*-value: 0.09, compared to the inactivated platform (**Table 2**).

The age distribution indicated that 43.5%, 32,6%, and 23.9% of cases were in the 40 to 70, 18 to 40, and older than 70 years age groups, respectively. Therefore, the mean age in the mortality group was 52.0 ± 19.6 [Median:55, IQR:32], not significantly different from the mean age of 47.0 ± 20.0 years [Median: 54, IQR:38], in 233 patients who recovered from related serious AEs (mean difference: 4.96, 95%CI: -1.38-11.3, *P*-value: 0.12). Moreover, the influence of age on the odds of survival showed an OR of 0.99, which was statistically insignificant (95%CI: 0.97-1.00, *P*-value 0.13).

Regarding sex, the mortality rate of 15.0% in men showed no significant disparity from the 18.2% mortality rate in women (OR: 0.82, 95%CI: 0.28-1.39, *P*-value: 0.52) (**Table 2**).

The mortality incidence index of these AEs per 100 000 vaccine injections was as follows: 0.03 in total, Razi-Cov-Pars 0.45, Sputnik V.0.14, Vaxzevria 0.14, PastoCovac 0.03, Covilo 0.02, and Barekat 0.01.

## 4 Discussion

Our study, conducted through Active and Passive Vaccine Safety Surveillance, spanned almost 2 years, from February 2021 to December 2022, and encompassed multiple variants of SARS-CoV2, including Beta, Delta, And Omicron. Among the 155 million COVID-19 vaccine doses administered in Iran, 279 related serious AEs were reported, resulting in a rate of 1.8 per million people (PMP). Covilo was the primary initial dose vaccine across all age groups, administered eight times more than Vaxzevria in total. In the 5-12-year age group, Covilo was injected 4 times more frequently than PastoCovac, and Covilo accounted for up to 97.5% of cases in the 12-18-year age group. For individuals aged 18-40 years, Covilo surpassed Vaxzevria by 10-fold. This ratio was approximately 7.5 times for those aged between 40-70 and 2.5 times for those over 70 years.

AEs mainly occurred after the initial dose. Most of the AEs (92.7%) were considered non-serious, predominantly manifesting as mild fever and localized symptoms. Regarding the incidence of serious AEs per 10,000 doses for different vaccine types, from highest to lowest, were Sputnik V, Vaxzevria, Barekat, and Covilo, with Sputnik V and Vaxzevria nearly double the rate compared to Covilo.

Coagulation and thrombosis disorders as well as neurological issues were predominant serious AEs. Sputnik V, Vaxzevria, and Razi-Cov-Pars vaccines exhibited incidence rates that were 4.8, 3.6, and 2.5 times higher than the average incidence per dose, respectively. Coagulation and thrombosis disorders, accounting for 81 cases (0.5 PMP), were mainly associated with Vaxzevria and Sputnik V, occurring at rates 4.8 and 4.4 times the average, respectively. Neurological issues accounting for 69 cases (0.4 PMP), with Sputnik V and Vaxzevria exhibiting rates 14.5 and 2.5 times the average, respectively. Relevant cardiovascular AEs, with a total of 44 cases (0.28 PMP), were mainly linked to Razi-Cov-Pars (16 times the average), Sputnik V (2.3 times), Vaxzevria (2 times), and Barekat (1.6 times). Cerebrovascular accidents, constituting 31 cases (0.2 PMP), were primarily associated with Vaxzevria (3.5 times the average). Other 54 cases (0.34 PMP), were also primarily related to Vaxzevria.

Non-thrombotic issues predominantly emerged after the first dose and reduced in subsequent administrations. Sex disparity was minor, and the age group of 40-70 years was the most affected. Most of the non-thrombotic AEs were associated with the Adenoviral platform. Ultimately, 93% of non-thrombotic cases showed improvement.

Thrombotic AEs were mostly observed after the first dose, with a slight preference for men, one-third of whom recovered. The thrombotic incidence was 5, 3.7, and 3.2 times higher than the average in Razi-Cov-Pars, Vaxzevria, and Sputnik V, respectively.

In Iran, with nearly 85 million population, 2.5 years after the beginning of COVID-19 vaccination (by June 2023) over 155 million doses of different vaccine types were administered to 65.9 million people, covering 74% of the population. Globally, 13 billion doses have been injected, with approximately 70.3% of the worldwide population receiving at least one dose. The vaccination guidelines in Iran targeted healthcare workers, high-risk groups, and individuals over 5 years old. Additionally, Covilo and PastoCovac are approved for ages 5-18, and Covilo and Vaxzevria are permitted for use in pregnant women (1, 19). In the US, vaccination began in December 2020 for healthcare workers and individuals over 6 months old are eligible to receive Moderna or Comirnaty (Pfizer-BioNTech) vaccines (20). In the UK, vaccination efforts started with healthcare staff and later extended to include all adults, including pregnant women and children above 5 years old. The UK predominantly used Moderna and Comirnaty vaccines (21).

In Saudi Arabia, a study involving 455 individuals was conducted over 11 days, utilizing a Google Form questionnaire on background and vaccine-related data shared via social media. However, the outcomes of this study are limited by the short duration and cannot determine long-term adverse effects (22). A United Arab Emirates cross-sectional study employing an online Google Form survey through social media platforms and telephone interviews focused on side effects occurring after vaccination. The study covered 744 people for 6 months, who were asked to rate side effects on a Likert scale of 1-10. Since the results were driven from a survey-based evaluation, determining causality is not possible (23). Another survey study, conducted in six different Arab countries, evaluated AEs among 1,564 people. The study observed that most local AEs were associated with Covilo vaccine, most systematic AEs with Comirnaty, and most serious AEs with the Jcovden (Johnson & Johnson) vaccine (24). An observational study in the USA analyzed data from the Vaccine Adverse Event Reporting System (VAERS) and V-safe, spanning 6 months in 2021. The VAERS reports were categorized as non-serious, serious, or death. Simultaneously, the V-safe survey reports were scrutinized within 0-7 days post-vaccination for factors such as reactogenicity, severity, and health impacts. The VAERS classified reports as serious based on outcomes such as hospitalization, prolonged hospitalization, permanent disability, life-threatening illness, congenital anomaly or birth defect, and death (9).

In Iran early preference for the Sputnik V and Covaxin vaccines for vulnerable groups may have contributed to higher AEs reports, as these groups received heightened attention in the reporting system. Additionally, as vaccination in Iran began in February 2021 (25), and the country experienced 2 waves of COVID-19 in the 6 months following the start of vaccination (26), it is highly likely that many individuals were infected or carriers at the time of vaccination. This is especially true since a COVID-19-negative PCR was not mandatory for vaccination, similar to the WHO and the Centers for Disease Control and Prevention (CDC) recommendations. Therefore, it is challenging to distinguish whether AEs were caused by the virus or the vaccine. Conversely, thrombosis events occur annually at an approximate incidence of 1 per 1 000 adults (27). Interestingly, COVID-19 patients initially reported a thrombosis prevalence of 22%, which increased to 43% for patients admitted to the intensive care unit (ICU) (28). These data suggest a significant protective effect of vaccines against these events.

Females (52.2%) exhibited higher mortality rates than males, and the 40-70-year age group had a higher mortality rate. Thrombotic AEs were 2.8 times more frequent than non-thrombotic issues as the cause of death post-vaccination.

Razi-Cov-Pars vaccines showed a 15 times higher mortality rate than the average, while Sputnik V and Vaxzevria were 4.6 times higher. Due to the lack of post-mortem examinations, there is no definite evidence linking these casualties to the vaccine. A German study investigated 18 post-mortem cases after vaccination. Comprehensive autopsies, histopathological analyses, and virological assessments were conducted in all instances. Subsequently, evaluations indicated that vaccine-induced immune thrombocytopenic thrombotic AEs was the cause of death in only 2 cases. This study, along with a similar study conducted in Japan, highlights the importance of post-mortem investigations for all deaths following vaccination to establish potential links between vaccination and mortality (29, 30).

In a previous nationwide study conducted in Iran, we reported that vaccination not only prevents COVID-19 disease but also reduces the risk of mortality by 25% in fully vaccinated individuals who become infected. Additionally, we observed that vaccination reduces ICU mortality. In this regard, compared to unvaccinated patients, the first, second, and third doses of the vaccine led to 15%, 19%, and 26% lower risk of ICU death, respectively. Additionally, the intubation rate and ICU stay were significantly reduced in the vaccinated group (25). Therefore, the positive effects of vaccination, as reported in prior studies, along with the extremely rare incidence of serious AEs following vaccination, underscore the significant role that vaccination plays in saving the lives. In another assessment on the cost-benefit of COVID19 vaccination in Iran, we determined that the vaccination program resulted in an average gain of 0.3 quality-adjusted life years (QALYs) per person. This improvement in health came at an additional cost of $14.08 per person. Overall, the program was highly cost-effective, with an average Incremental Cost-Effectiveness Ratio (ICER) of $406.85 across the adult population. This means the benefits of vaccination far outweigh the costs compared to not vaccinating (unpublished data).

The prospective design of our study, along with the comprehensive method of assessing AEs—first in the hospital, then in university committees, and finally at the national committee by various specialists—are the main strengths of our study. Additionally, each AE (especially serious AEs) was evaluated at the time of occurrence by physicians, unlike most previous studies, which relied on surveys and asked participants about post-vaccination AEs retrospectively. Furthermore, to the best of our knowledge, this study is one of the very few that evaluates all post-vaccination AEs across an entire nation and encompasses a very large sample size.

However, our study has some limitations. There is a possibility of underreporting and underestimating AEs, particularly non-serious AEs, which limits the study’s ability to capture the full spectrum of findings. Additionally, there is a lack of postmortem autopsies for vaccine-related mortalities in our study. Another limitation of this study the absence of data on the comorbidities of vaccinated patients.

Overall, we recommend a number of significant advancements to increase our knowledge of COVID-19 vaccine. First, by indicating variations in healthcare systems, immunization practices, and demographics, incorporating data on adverse occurrences from around the world would provide insightful context. Second, evaluating AEs patterns from diverse vaccination platforms—including mRNA vaccines—according our method, will give crucial information about how safe various vaccine technologies are. Third, we suggest using results from international autopsy reports and carrying out long-term follow-up studies to prove causation and detect any potential delayed AEs in order to overcome the shortcomings of the existing research and manage the possible outbreaks in the world.

## 5 Conclusion

The adverse events of COVID-19 vaccinations, such as flu-like symptoms, low-grade fever, localized reactions, and joint discomfort have generally been mild and temporary. It is worth noting that Sputnik V, Vaxzevria, Barekat, and Covilo vaccines exhibit a higher incidence of these symptoms. Importantly, serious AEs linked to the vaccines are exceedingly rare, underscoring the life-saving efficacy of these vaccines. Definitive evidence supports the vaccines’ effectiveness in preventing severe illness, hospitalization, and death. We believe that efforts should focus on emphasizing the beneficial impacts of vaccination, accompanied by transparent reporting of vaccine adverse events (AEs). Such transparency is essential to maintaining public trust.

## Data Availability

The raw data supporting the conclusions of this article will be made available by the authors, without undue reservation.
